# Implementation of Patient-Centered Care by Athletic Training Students during Clinical Experiences: A Report from the Association of Athletic Training Education Research Network

**DOI:** 10.3390/ijerph20085513

**Published:** 2023-04-14

**Authors:** Julie M. Cavallario, Bonnie L. Van Lunen, Stacy E. Walker, R. Curtis Bay, Cailee E. Welch Bacon

**Affiliations:** 1 School of Rehabilitation Sciences, College of Health Sciences, Old Dominion University, Norfolk, VA 23529, USA; 2 College of Health Sciences, Old Dominion University, Norfolk, VA 23529, USA; 3 School of Kinesiology, Ball State University, Muncie, IN 47306, USA; 4 Department of Interdisciplinary Health Sciences, Arizona School of Health Sciences, A.T. Still University, Mesa, AZ 85206, USA; 5 Department of Athletic Training, Arizona School of Health Sciences, A.T. Still University, Mesa, AZ 85206, USA; 6 Department of Basic Medical Science, School of Osteopathic Medicine in Arizona, A.T. Still University, Mesa, AZ 85206, USA

**Keywords:** goals, communication, informatics, student role, preceptor

## Abstract

Patient-centered care (PCC) is a core competency that should be required by all healthcare education programs, but little is known about its implementation in athletic training clinical experiences. Therefore, we examined characteristics of patient encounters documented by athletic training students implementing PCC behaviors. A multisite panel design was used to recruit 363 students from twelve professional athletic training programs (five undergraduate, seven graduate). Over 1.5 years, clinical experience patient encounter data were logged in E*Value Case Logs, including student role during the encounter, length of encounter, and clinical site. Generalized estimating equations models characterized the likelihood students included PCC behaviors in 30,522 encounters. Discussing patient goals was associated with student role (χ^2^(2) = 40.6, *p* < 0.001) and length of encounter (χ^2^(4) = 67.6, *p* < 0.001). Using patient-reported outcome measures was associated with student role (χ^2^(2) = 21.6, *p* < 0.001), length of encounter (χ^2^(4) = 34.5, *p* < 0.001), and clinical site (χ^2^(3) = 17.3, *p* = 0.001). Implementing clinician-rated outcome measures was affected by length of encounter (χ^2^(4) = 27.9, *p* < 0.001) and clinical site (χ^2^(3) = 8.6, *p* = 0.04). PCC behaviors were largely associated with student role and length of encounters; clinical site had less impact. Athletic training educators should emphasize progressive autonomous supervision with preceptors and encourage students to facilitate slightly longer patient visits, when possible, to incorporate more PCC behaviors.

## 1. Introduction

**Figure 1:**
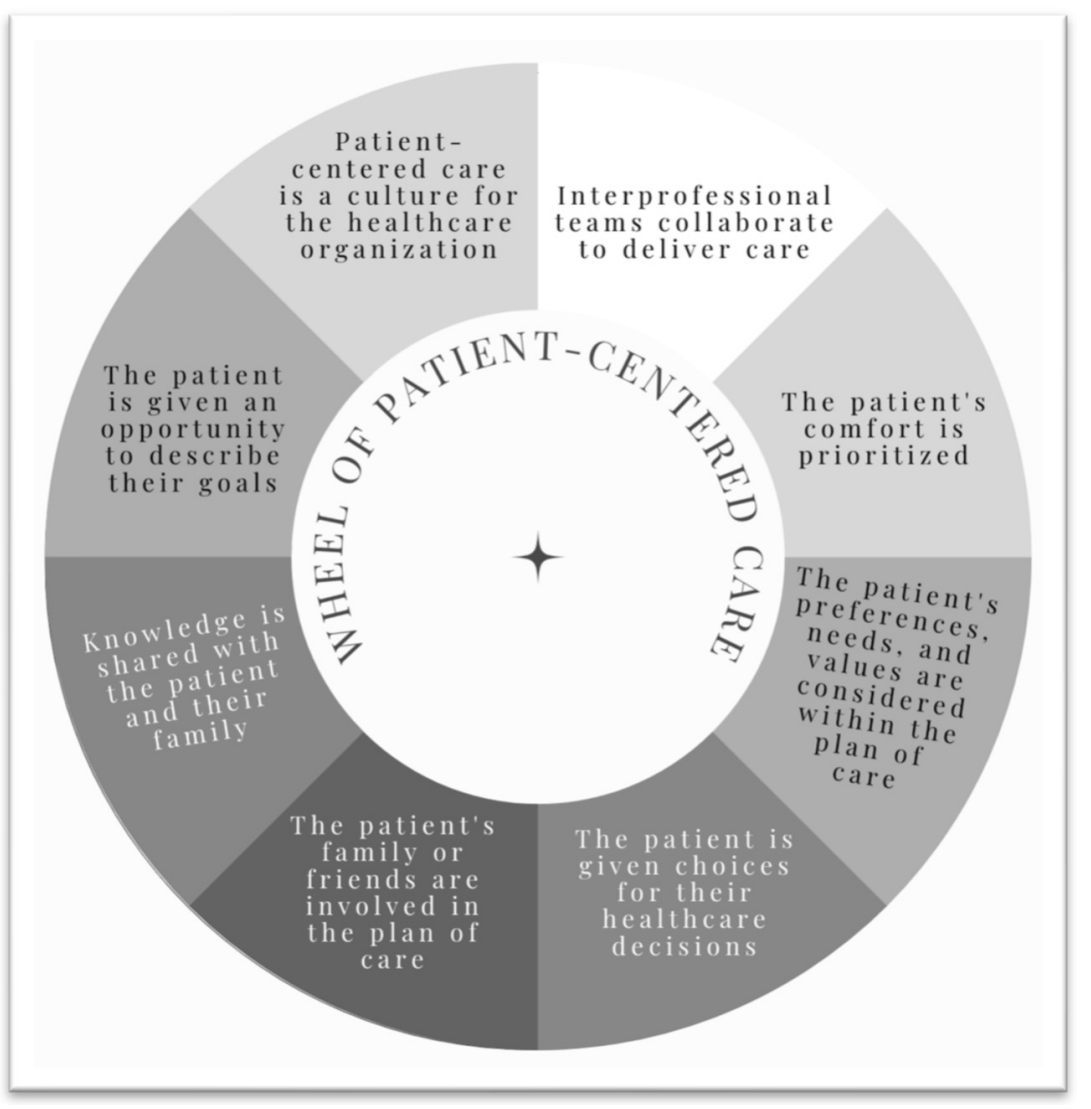
Wheel of patient-centered care [1,3,4,5,6].

More than 20 years ago, the Institute of Medicine identified patient-centered care (PCC) as one of the essential components of quality healthcare [1,2,3]. Although varying definitions of PCC exist [1,3,4,5,6], most include transparency, shared decision-making, incorporation of the patient’s goals and preferences, and prioritization of patient comfort. Figure 1 presents the essential components of PCC. Despite recommendations to incorporate PCC and the other core competencies into the provision of all healthcare services [3], it has taken many years for healthcare education programs and healthcare delivery systems to embrace PCC as an integral component of high-quality healthcare, and adoption of PCC has come with varying levels of success [7,8,9].

In athletic training education, PCC was first introduced in the advanced practice landscape, with requirements to incorporate it into post-professional degree and residency programs in 2014 [10], and became a required component of entry-level graduate programs in 2020 [11]. Consistent with other healthcare education programs, research conducted on athletic training students in professional programs before the adoption of PCC has shown that PCC is the core competency that students most often believe they are implementing [8,10,12]. Researchers have also reported that, although athletic training students most often attempt to ask the patient about their goals when implementing PCC, they omit PCC behaviors in more than 40% of all patient encounters (PEs) [12].

To increase the implementation of PCC, we need a greater understanding of the characteristics of individual patient interactions that facilitate consistent PCC behaviors. Therefore, the purpose of the current study was to examine the characteristics of PEs documented by athletic training students implementing PCC behaviors.

## 2. Materials and Methods

### 2.1. Design

A multisite panel design was used to track the PEs of athletic training students during their clinical experiences. Data from 12 athletic training programs were collected for 1.5 academic years. All PEs were entered in the E*Value Case Logs software, which is a web-based data management system (MedHub, Minneapolis, MN, USA). Before data collection, institutional review board approval was granted by the sponsoring institutions and, as needed, the participating institutions. Within the United States of America, athletic training education is a healthcare education program that prepares students to engage in patient care with physically active individuals across five domains of practice: (1) Risk Reduction, Wellness, and Health Literacy; (2) Assessment, Evaluation, and Diagnosis; (3) Critical Incident Management; (4) Therapeutic Interventions; and (5) Healthcare Administration and Professional Responsibility. Students within such programs engage in clinical experiences in which they participate in actual patient care under the supervision of a preceptor, otherwise known as a clinical instructor or clinical supervisor. Educational programs may choose to use software programs, such as E*Value, to have students log the patient cases they experience during clinical experiences to use for program and student assessment.

### 2.2. Participants

We recruited Commission on Accreditation of Athletic Training Education (CAATE)-accredited professional athletic training programs by contacting the program directors and clinical education coordinators of each program to determine interest and eligibility to participate. Our inclusion criteria for participation consisted of the following: (1) the program used E*Value to track student PEs during clinical experiences for at least 1 year before the study, (2) the program required students to track PEs using the Case Logs module in E*Value during clinical experiences, and (3) the program had a 3-year aggregate Board of Certification first-time pass rate greater than 85% [12]. Twelve of the fifteen programs that met all the inclusion criteria agreed to participate in the study. During the study, 5 of the 12 professional athletic training programs were at the bachelor’s degree level; the other 7 were at the master’s degree level.

Students enrolled in the participating programs (*n* = 363) were informed that their program had chosen to participate in the study and that informed consent forms had been completed by the program director. Since all PEs recorded by the students were a required part of their program’s organized clinical experiences, obtaining informed consent from individual students was deemed unnecessary. Before data collection, one member of the research team worked with the program director of each program to confirm that the E*Value Case Logs module included all the pertinent data fields [12].

### 2.3. Instrumentation

Data for the current study were collected using E*Value Case Logs. The Case Logs module allows students to securely record data specific to PEs from their assigned clinical experiences. For each PE, students log several data points, such as patient procedural opportunities, PE characteristics, student role during the PE, and student engagement with core competency-related professional behaviors. The operational definition of a PE, an interaction with a patient in which the patient’s current functional status or a plan for future care was discussed with the patient, was also provided to students prior to the implementation of data collection. Details regarding the specific variables collected and the development and validation of the core competency-related professional behaviors have been published previously [12,13,14]. For the purposes of the current study, we focused only on the professional behaviors related to PCC core competency. A list of the professional behaviors identified for PCC is provided in Table 1. In the case of the student observing their preceptor during the PE, students were instructed during the programmatic training sessions to indicate that the professional behavior associated with PCC was included whether they themselves performed the behavior or if they observed their preceptor doing so.

### 2.4. Procedures

Before data collection, a member of the research team conducted training sessions about program- and student-level study procedures. After training, students from all participating programs were instructed by their program faculty to log all PEs they engaged with during their clinical experiences. To ensure data quality was maintained throughout the study, data were downloaded by each program and securely transferred to the research team every 2 weeks. Data collection for 3 of the 12 programs began during the spring 2018 semester; we initiated data collection with a limited number of programs to ensure study procedures and processes were in place. The remaining 9 programs began data collection during the fall 2018 semester, and data collection for all 12 programs continued through the end of the spring 2019 semester. Since no changes to study procedures or processes were made at the end of the spring 2018 semester, data collected from the 3 initial programs were included in our analyses, so the total data collection duration was 1.5 academic years. After conclusion of the study (spring 2019), each participating program received a research study honorarium. A detailed description of the study procedures, including program and student-level training procedures, has been published elsewhere [12].

### 2.5. Data Analysis

We used descriptive statistics, including frequencies (percentages) and means (standard deviations), to summarize PCC behaviors performed by students during PEs. Generalized estimating equations models (GEE) with Poisson distributions and robust covariance estimators were used to characterize the likelihood (relative risk ratios) that students included the following at each encounter: (1) discussed patient goals with the patient, (2) collected information using a patient-reported outcome measure (PROM), (3) collected information using a clinician-rated outcome measure (CROM), and (4) implemented no PCC behaviors. Potential predictors (PE characteristics) included student role (performed, assisted, observed), length of encounter (1 min to >60 min), and type of clinical site (college/university, high school, clinic, other). An analogous GEE approach was employed to analyze the association of PE characteristics on the composite opportunities (ranging from none of the behaviors to all 3 of the behaviors, 0–3 behaviors) for all PCC behaviors that students included during PEs. Only predictors that significantly contributed to the equation were retained in the final model. Sequential Bonferroni corrections were applied to pairwise comparisons. An a priori significance threshold of α < 0.05 was set for all analyses. Data were analyzed using SPSS version 27 (IBM Corp., Armonk, NY, USA).

## 3. Results

For the 12 participating programs, 30,522 PEs (99.6% of all recorded PEs, *n* = 30,630) included a response for PCC behaviors documented by 338 athletic training students. Descriptive information about the participating programs has already been published elsewhere [12]. Relative to each individual behavior, students discussed the patient’s goals with the patient in 39.5% of PEs, used a PROM in 30.1% of PEs, and used a CROM in 14.4% of PEs. Students implemented no PCC behaviors in 43.4% of PEs.

### 3.1. Discussing the Patient’s Goals with the Patient

Student role (χ^2^(2) = 40.6, *p* < 0.001) and length of encounter (χ^2^(4) = 67.6, *p* < 0.001) were associated with whether the student discussed the patient’s goals with the patient. Students who performed the PE (*n* = 21,801 PEs) under the supervision of their preceptor (RR = 1.4; 95% CI, 1.28 to 1.61; *p* < 0.001) and those (*n* = 5052 PEs) who assisted their preceptor throughout the PE (RR = 1.4; 95% CI, 1.22 to 1.50; *p* < 0.001) were more likely to discuss the patient’s goals with the patient than those who observed their preceptor completing the PE (*n* = 3669 PEs). When the length of PEs was 31–45 min (*n* = 2328 PEs), students were more likely to discuss the patient’s goals (RR = 1.6; 95% CI, 1.40 to 1.74; *p* < 0.001) than in PEs of 1–15 min (*n* = 18,019 PEs). When compared with PEs of 1–15 min, PEs of 16–30 min (*n* = 8819 PEs; RR = 1.3; 95% CI, 1.18 to 1.43; *p* < 0.001), PEs of 46–60 min (*n* = 904 PEs; RR = 1.5; 95% CI, 1.24 to 1.80; *p* < 0.001), and PEs longer than 60 min (*n* = 452 PEs; RR = 1.5; 95% CI, 1.23 to 1.71; *p* < 0.001) were more likely to include this professional behavior.

### 3.2. Use of Patient-Reported Outcome Measures

The use of PROMs by students was associated with student role (χ^2^(2) = 21.6, *p <* 0.001), length of the encounter (χ^2^(4) = 34.5, *p* < 0.001), and type of clinical site (χ^2^(3) = 17.3, *p* = 0.001). When performing the PE under the supervision of their preceptor (RR = 1.8; 95% CI, 1.39 to 2.27; *p* < 0.001) and when assisting their preceptor during the PE (RR = 1.6; 95% CI, 1.30 to 2.02; *p* < 0.001), students were more likely to use a PROM than when observing their preceptor perform the PE. When compared with PEs of 1–15 min, students were more likely to use a PROM in PEs of 16–30 min (RR = 1.3; 95% CI, 1.16 to 1.43; *p* < 0.001), PEs of 31–45 min (RR = 1.5; 95% CI, 1.29 to 1.74; *p* < 0.001), PEs of 46–60 min (RR = 1.5; 95% CI, 1.22 to 1.78; *p* < 0.001), and PEs longer than 60 min (RR = 1.4; 95% CI, 1.09 to 1.71; *p* = 0.007).

For type of clinical site, no differences were found between college/university (*n* = 20,016 PEs) and high school (*n* = 8307 PEs) sites relative to PROM use (*p* = 0.12). However, when compared with college/university sites, students who performed the PE at another site were less likely to use a PROM (RR = 0.8; 95% CI, 0.64 to 0.93; *p* = 0.008), and those who performed the PE at clinical sites were more likely to use a PROM (RR = 1.3; 95% CI, 1.09 to 1.67; *p* = 0.006).

### 3.3. Use of Clinician-Rated Outcome Measures

Student use of CROMs during PEs was associated with length of the encounter (χ^2^(4) = 27.9, *p* < 0.001) and type of clinical site (χ^2^(3) = 8.6, *p* = 0.04). When compared with PEs of 1–15 min, students were more likely to use a CROM in PEs of 16–30min (RR = 1.4; 95% CI, 1.19 to 1.70; *p* < 0.001), PEs of 31–45 min (RR = 1.4; 95% CI, 1.15 to 1.75; *p* = 0.001), PEs of 46–60 min (RR = 1.6; 95% CI, 1.18 to 2.28; *p* = 0.003), and PEs longer than 60 min (RR = 1.8; 95% CI, 1.42 to 2.29; *p* < 0.001). Relative to type of site, PEs performed at clinical sites were more likely to involve a CROM than those at college/university sites (RR = 1.4; 95% CI, 1.10 to 1.88; *p* = 0.008).

### 3.4. Implemented No Patient-Centered Care Behaviors

Similar to discussing patient goals with the patient, the likelihood that students implemented no PCC behaviors was associated with student role (χ^2^(2) = 43.6, *p* < 0.001) and length of the encounter (χ^2^(4) = 53.9, *p* < 0.001). Students who performed the PE (RR = 0.7; 95% CI, 0.63 to 0.79; *p* < 0.001) or assisted their preceptor with the PE (RR = 0.7; 95% CI, 0.63 to 0.78; *p <* 0.001) were less likely to implement no PCC behaviors than those who observed the PE. When compared with PEs of 1–15 min, students were less likely to implement no PCC behaviors in PEs of 16–30 min (RR = 0.8; 95% CI, 0.69 to 0.82; *p* < 0.001), PEs of 31–45 min (RR = 0.6; 95% CI, 0.56 to 0.73; *p* < 0.001), PEs of 46–60 min (RR = 0.68; 95% CI, 0.52 to 0.88; *p* = 0.003), and PEs longer than 60 min (RR = 0.82; 95% CI, 0.71 to 0.94; *p* = 0.005).

### 3.5. Composite Patient-Centered Care Behaviors

The overall clinical associations of PCC behaviors and associated characteristics of documented PEs are presented in Table 2. The total number of PCC behaviors included in a PE were affected by student role (χ^2^(4) = 30.0, *p* < 0.001) and length of the encounter (χ^2^(4) = 111.1, *p* < 0.001). Students who performed the PE (RR = 1.4; 95% CI, 1.24 to 1.63; *p* < 0.001) or assisted their preceptor with the PE (RR = 1.4; 95% CI, 1.23 to 1.55; *p* < 0.001) were more likely to implement more PCC behaviors than those who observed the PE. When compared with PEs of 1–15 min, students were likely to implement more PCC behaviors during PEs of 16–30 min (RR = 1.3; 95% CI, 1.22 to 1.42; *p* < 0.001), PEs of 31–45 min (RR = 1.6; 95% CI, 1.43 to 1.70; *p* < 0.001), PEs of 46–60 min (RR = 1.5; 95% CI, 1.30 to 1.69; *p* < 0.001), and PEs longer than 60 min (RR = 1.5; 95% CI, 1.26 to 1.70; *p* < 0.001).

## 4. Discussion

Although the overall project collected a wide variety of variables to represent the characteristics of PEs, in the current study, only three variables were associated with the use of PCC behaviors by athletic training students: student role during the PE, length of the encounter, and type of clinical site where the PE occurred.

### 4.1. Student Role

Our findings for student role during PEs suggested that increased student autonomy during their clinical experiences resulted in an increase in PCC behaviors. Students who performed the PE were the most likely to ask the patient about their goals, use a PROM, and include more PCC behaviors overall. This finding slightly conflicts with previous research in athletic training education that reported students who assisted their preceptor during PEs had the greatest odds of implementing PCC [10]. In our study, students who assisted their preceptor during the PE had an equal relative risk of asking the patient about their goals or implementing multiple PCC behaviors as students who performed the PE, but those who assisted did not have as high of an increased likelihood of using a PROM.

A participatory action research study conducted in family medicine education determined that clinical supervisors must intentionally introduce and describe core competencies during patient interactions in order for students to embrace their use in clinical practice [15]. Similarly, our findings indicated that students who assisted their preceptor during PEs had increased implementation of PCC. Perhaps those who implemented PCC more frequently when performing PEs had previously been instructed on its use during earlier clinical rotations and, therefore, felt empowered to implement those behaviors autonomously. However, we are unable to verify this supposition.

Students who observed their preceptor during PEs were the most likely to indicate that no PCC behaviors were implemented during encounters. Researchers have previously reported that observation of working clinicians, especially when working in interprofessional teams, gives students the opportunity to reflect on attitudes to other healthcare professions, the power dynamics between clinicians and patients and between clinicians and other healthcare professionals, and the influence of communication on patient care [16]. One suggestion stemming from this research would be for athletic training educators to provide training to students that would allow them to engage in active observation and reflection, thus making observed PEs more meaningful. Such training could also improve the perceived implementation of PCC behaviors during observed experiences, possibly leading to improvements in the actual implementation of those behaviors when students are in a more autonomous role.

### 4.2. Length of Encounter

The amount of time spent with the patient was associated with all PCC behaviors, including the likelihood of implementing no PCC behaviors or the greatest number of PCC behaviors. The PEs that lasted 31–45 min seemed to hit the metaphorical sweet spot for PCC; this length of encounter had the greatest likelihood of students discussing the patient’s goals with the patient, using a PROM, and implementing more PCC behaviors. This length of time also had the lowest likelihood of students implementing no PCC behaviors during PEs. This finding was somewhat consistent with previous research in athletic training education that reported longer PEs had an increased odds ratio of implementing PCC [10]. However, the authors of that study [10] used a dichotomous approach to evaluate whether PCC was being implemented or not, and they did not examine individual behaviors associated with PCC competency.

In ambulatory healthcare practice settings that employ athletic trainers, average patient visit times were 31 min for new patients and 19 min for established patients [17]. Although this structure appears to facilitate the inclusion of PCC behaviors, especially during new patient appointments, this length of time for a patient appointment may not be ideal for those in traditional athletic training practice settings, such as high schools or colleges. In those settings, patients are in class for much of the day and typically arrive for treatment before scheduled practice or competitions. In such cases, the majority of visits are less than 15 min [13].

Importantly, the length of the patient interaction influences much more than just the use of PCC behaviors, as measured in the current study. Shorter patient visits have been associated with decreases in patient and clinician satisfaction, poorer patient outcomes with chronic disease treatment, and an increase in malpractice claims [18]. To improve PCC implementation when there is a limited time frame, Stewart et al. [19] recommended that clinicians adopt a strategy for patient-centered communication that includes (1) preparing an agenda for each visit to ensure a focused interaction, (2) tuning in to the patient’s emotional agenda and responding accordingly, (3) employing active listening and empathic responses, (4) encouraging patient participation and presentation of their condition from their viewpoint, and (5) agreeing on goals for treatment and future visits. Athletic training program faculty should consider incorporating patient-centered communication into athletic training curricula and encourage its use during clinical experiences. Such changes may improve the implementation of PCC during the shorter patient visits that are common in traditional athletic training practice settings.

### 4.3. Type of Clinical Site

Although not as consistently related to PCC behaviors as student role or length of encounter, the type of clinical site where the student was interacting with patients had some association with the use of PROMs and CROMs. This finding was consistent with the literature in athletic training education that indicated the clinical site had minimal influence on the use of PCC behaviors during PEs; however, differences in that study were found between college and high school settings [10], which was not the case in our study. Unfortunately, the overall evidence in athletic training education is lacking regarding the use of PCC based on type of clinical site.

In medical education, students who spent an entire year at one clinical site had less degradation of PCC behaviors and attitudes than those who attended multiple clinical rotations during the year [20]. However, the study had a small participant pool [20], so it should be interpreted cautiously. Despite those limitations, athletic training program administrators should consider instituting longer placements at clinical sites to make students more comfortable with implementing PCC behaviors.

Other researchers have reported that implementation of PCC behaviors may have less to do with site type and more to do with the informatics infrastructure of the site [21]. For example, sites that invest in electronic health records to communicate between providers and that promote the use of PROMs and CROMs, encrypted email, and patient portal options allow patients to communicate with providers [21]. These sites also typically have telehealth options that decrease patient burden when seeking medical guidance [21]. In addition to the culture of informatics at a given site, other researchers have suggested that the culture of communication within a clinical site may have significant influence over the patient-centeredness of care and communication by providers and students within the site [22,23]. Given the variability of findings for the effect of the clinical site on PCC implementation in athletic training education, we encourage athletic training educators to analyze the availability of informatics and the culture of communication at the different sites used for student clinical experiences. For sites that have limited informatics options, programs could augment the site infrastructure with externally hosted academic electronic health records. If the site has limited availability of informatics and PCC opportunities, programs should reconsider whether to continue using that site for student education.

### 4.4. Limitations and Future Research

The strength of this study is the longitudinal nature of examining PEs over a year of time across different athletic training programs. Another strength that built upon the existing body of evidence was the identification of behaviors associated with PCC to increase the specificity of which aspects of PCC were being used. A limitation of our study was that we only examined aspects of PCC behaviors that could be implemented during PEs. We did not examine the role of organizational culture or other institution-wide contributors on these PCC behaviors in the healthcare system. The data collection process also relied on the participating athletic training students accurately self-reporting their behaviors during PEs. Future research should examine the interrelatedness of PCC and informatics infrastructure at various clinical sites to characterize the relationship of PCC and informatics more accurately. Future research should also aim to evaluate how students are assessed on the implementation of patient-centered care by preceptors or programmatic faculty. If a similar study is implemented in the future, researchers should aim to triangulate student perceptions with preceptor observations to strengthen the accuracy of the findings.

## 5. Conclusions

In the current study, student role and length of the encounter had an overall greater association with the implementation of PCC behaviors during PEs, and the type of clinical site had a minimal role on the implementation of PCC behaviors. However, our clinical site findings may have been influenced more by the informatics infrastructure of the site rather than the type of site. Therefore, athletic training education programs should evaluate the informatics available at clinical sites to identify opportunities for increasing student proficiency in both PCC behaviors and healthcare informatics. According to our results, as autonomy increased, students were more likely to ask the patient about their goals and use PROMs. As the length of the encounter increased, there was a general increase in overall PCC behaviors; the optimal length of time for implementing PCC behaviors during a PE was 31–45 min. For program administrators hoping to increase the PCC behaviors of their students during clinical rotations, clinical site data should be analyzed to determine which sites promote greater patient autonomy and have average PE lengths of 31–45 min. Alternatively, program faculty should focus on patient-centered communication to increase the likelihood that PCC behaviors are implemented during shorter PEs or that preceptors have increased supervision and involvement.

## Figures and Tables

**Table 1:** Professional behaviors performed for PCC core competency.

Professional Behavior ^1^
Discuss the patient’s goals with the patient?
Collect information through a patient-reported outcome measure?
Collect information through a clinician-reported outcome measure?
None of the above.

^1^ For each prospective professional behavior that could have been included in the PE, the response was yes or no.

**Table 2:** Clinical associations of patient-centered care (PCC) behaviors implemented by athletic training students during patient encounters (PE) ^1^.

PE Characteristic	PCC Behavior				
	Discussed Patient Goals with Patient	Use of Patient-Reported Outcome Measure	Use of Clinician-Rated Outcome Measure	Implemented No PCC Behaviors	Composite PCC Behaviors
Student role					
Performed	**40% more likely**	**80% more likely**	not significant	30% less likely	**40% more likely to have more behaviors**
Assisted	**40% more likely**	60% more likely	not significant	30% less likely	**40% more likely to have more behaviors**
Observed	comparison variable	comparison variable	not significant	**comparison variable**	comparison variable
Length of encounter					
1–15 min	comparison variable	comparison variable	comparison variable	**comparison variable**	comparison variable
16–30 min	30% more likely	30% more likely	40% more likely	20% less likely	30% more likely to have more behaviors
31–45 min	**56% more likely**	**50% more likely**	60% more likely	40% less likely	**60% more likely to have more behaviors**
46–60 min	50% more likely	**50% more likely**	40% more likely	32% less likely	50% more likely to have more behaviors
>60 min	50% more likely	40% more likely	**80% more likely**	18% less likely	50% more likely to have more behaviors
Type of clinical site					
College/university	not significant	comparison variable	comparison variable	not significant	not significant
High school	not significant	not significant	not significant	not significant	not significant
Clinic	not significant	20% less likely	**40% more likely**	not significant	not significant
Other	not significant	**30% more likely**	not significant	not significant	not significant

^1^ Bolding represents the characteristics of the patient encounter that students were most likely to include.

## Data Availability

Data from this study are not publicly available due to privacy restrictions.
